# Association of Baldness with Coronary Artery Disease and Its Severity

**DOI:** 10.31661/gmj.v9i0.1474

**Published:** 2020-10-03

**Authors:** Reza Arefi, Mehdi Pishgahi, Adel Joharimoghaddam, Mohammad Ali Momeni, Mehran Khoshfetrat

**Affiliations:** ^1^Research Committee of AJA University of Medical Science, Tehran, Iran; ^2^Cardiovascular Research Center, Shohadaye Tajrish, Shahid Beheshti Medical Science University, Tehran, Iran

**Keywords:** Coronary Artery Disease, Baldness, Alopecia

## Abstract

**Background:**

The pivotal role of baldness as a potential risk factor for cardiovascular disorders remains a debate, and the small body of literature has generated inconsistent findings. We aimed to assess the association between baldness and the risk for coronary artery disease (CAD) and its severity in a sample of Iranian men.

**Materials and Methods:**

This cross-sectional study was performed on 105 consecutive patients suspected to CAD and scheduled for elective coronary angiography. The severity of CAD was determined according to the number of involved coronary vessels. For assessing the severity of baldness, the Hamilton-Norwood scale for grading of androgenetic alopecia in males was used.

**Results:**

The overall frequency of CAD in the groups with and without baldness was 88.9% and 72.5%, respectively, indicating a significant difference (P=0.033). Similarly, patients with the family history of baldness suffered more from CAD as compared to those without history (93.3% vs.76.0%). However, no significant association was found between the baldness and non-baldness groups with regard to the severity of CAD (P=0.291). According to the multivariable logistic regression model, the presence of baldness could predict the increased likelihood of CAD (or=3.037, P=0.046).

**Conclusion:**

Along with traditional risk factors for CAD, the presence of baldness and positive family history of this phenotype could be considered as the primary determinant for the increasing likelihood of CAD.

## Introduction

For many years, major genetic and environmental risk factors for cardiovascular disorders have been revealed, and in this regard, predicting programs and algorithms have been introduced to early diagnosis of such factors as well as to determine the likelihood of cardiovascular disorders. However, some reports have also emphasized the potential risk for these disorders in the lack of known risk factors [1-4]. In other words, known classic risk factors may not enough to cardiovascular risk assessment in suspected individuals. Thus, recently attempted focused on discovering new risk factors. Male pattern baldness is also known as androgenic alopecia, is now suggested to be associated with several metabolic disorders [5-7]. Male pattern baldness may be the risk factor for cardiovascular disease was first suggested, in 1972, when Cotton et al. [8] indicated an association between cardiovascular diseases and hair loss. This polygenic phenomenon generally began at the second or third decade and is identified as varying grades of hair loss. Some studies have suggested the relationship between baldness and increased risk for coronary artery disease (CAD) and its related risk factors [9,10]. Even the Framingham Heart Study [11] as a great global cohort on cardiovascular disorders, could indicate evidence on the association between the severity of hair loss within adulthood and the risk for CAD [11]. In some studies, baldness was shown to be associated with a 3-fold higher risk of myocardial infarction [12]. The pathophysiology of this causality remains unclear. It is now hypothesized that high androgen levels in people with baldness may predispose the vascular bed to be atherosclerotic and thrombotic events [13]. More interestingly, the pattern of baldness (i.e., vertex or frontal baldness) and its grade has been found to be associated with the risk for CAD [14]. However, the paucity of literature has generated inconsistent findings, and reviewing the literature reflects the major controversy. In this regard, the relation between baldness and CAD in Iranian men are unclear. Hence, we aimed for the first time to assess the association between baldness and other factors on CAD and its severity among Iranian men and demonstrated the precise effect of these factors on CAD.

## Materials and Methods

###  Patients

 This cross-sectional study was performed on 105 consecutive patients that all of them had Iranian race and suspected to CAD and scheduled for elective coronary angiography that referred to Imam Reza and Shahid Modarres hospital in Tehran, Iran during 2019. The ethics committee of Iran University of Medical Sciences approved the study (IR.SBMU.MSP.REC.1398.369), and all participants gave written informed consent before participating and undergoing angiography.

###  Data Collection

Exclusion criteria were female sex, history of coronary artery bypass surgery, history of the acute coronary syndrome in the past four weeks, other types of alopecia, and patients with known hormonal problems. Baseline characteristics including demographic data, anthropometric parameters, medical history regarding classic CAD risk factors (such as diabetes mellitus, hypertension, smoking status, opium use, alcohol use, and family history of CAD) and also the history of baldness in the relatives were all collected by interviewing before coronary angiography. Before coronary arteriogram, the presence and severity of hair loss were recorded. For assessing the severity of baldness, this phenomenon was graded according to the Hamilton-Norwood scale for grading of androgenetic alopecia in males (grades I-VII) [15]. The subjects with baldness grades I-III were considered as the low-grade disease, and those with grades IV-VII were regarded as a high-grade disease. The severity of CAD was determined according to the number of involved coronary vessels (minimal CAD, one-vessel, two-vessel, and three-vessel disease) as well as the presence of left main lesions.

###  Statistical Analysis

 The study endpoint was to assess the association between the grade of baldness and the presence and severity of CAD. In this regard, descriptive analysis was used to describe the data, including mean and standard deviation (SD) for quantitative variables and frequency (percentage) for categorical variables. Chi-square test, independent t-test, and Mann-Whitney U test were used for comparison of variables. To determine the value of baldness to predict presence of CAD, the multivariable logistic regression analysis was employed. For the statistical analysis, the statistical software IBM SPSS Statistics for Windows version 22.0 (IBM Corp. Released 2013, Armonk, New York, USA) was used. P-values<0.05 were considered statistically significant.

## Results

 The mean age of participants was 58.45 ± 13.46 years (ranged 30 to 91 years), and the mean body mass index was 26.23 ± 2.66 kg/m2. Regarding occupational status, 59.0% had an army-dependent job status, 26.7% were employed, 13.3% were self-employed, and others were unemployed. Classic cardiovascular risk factors show in Table-1 that demonstrate being smoking and having hypertension are the important risk factors for CAD. The mean left ventricular ejection was 44.55 ± 11.09%, which 23.8% had ejection fraction lower than or equal to 35% (Table-2).

 According to Table-3, 44.4% and 55.6% of patients with baldness were stratified as low- and high-grade, respectively. As shown in Table-4, there was no significant difference in baseline variables, including occupational status, body mass index, and traditional cardiovascular risk factors across the people with and without baldness; however, the patients with baldness were older than those without baldness and family history was an important factor for patients with baldness. The overall prevalence of CAD in the groups with and without baldness was 88.9% and 72.5%, respectively, indicating a significant difference (P=0.033). Similarly, patients with the family history of baldness suffered more from CAD as compared to those without history (93.3% vs. 76.0%). However, no significant association was found between the baldness and non-baldness groups with regard to the severity of CAD (P=0.291, Table-5). We found no significant difference between the patients with and without CAD in the grade of baldness, but generally, CAD among patients with baldness was more than patients without baldness (P=0.204). No significant correlation was revealed between the number of involved coronary arteries and the grade of baldness (r=0.028, P=0.775). More importantly, the mean left ventricular ejection fraction was slightly lower in those patients with baldness as compared to groups without baldness (42.30±11.17% vs. 46.24 ± 12.09, P=0.046). According to the multivariable logistic regression model (Table-6), the presence of baldness could predict the increased likelihood of CAD by three times (r=3.037, P=0.046).

## Discussion

In line with some studies in the literature, we could show a close association between baldness and increased risk for CAD, even independent of the patients’ age and grade of baldness. In this regard, the presence of baldness in men might increase the likelihood of CAD by about 3-fold. Even adjusting potential powerful confounders and parameters including cardiovascular risk profile, the major role of baldness remains constant; however, we could not demonstrate an association between CAD severity and grade of balding, but generally, baldness increases the risk of CAD. Although the Hamilton-Norwood scaling is a usual classification for balding, this scaling method was modified many times. Moreover, we only used the number of involved coronary vessels as a variable for CAD severity that may not be a good indicator of CAD severity. In this regard, several studies used the criteria, which considered the extension of coronary atherosclerosis, such as Gensini score [16]. In total, some studies could find similar findings, and some others expressed contradictory results. In a study by Sari et al. [16], patients with baldness had a higher Gensini score when compared with their non-bald counterparts; however, in their multivariate analysis, only older age, obesity, and smoking were independent predictors of a high Gensini score. We showed that smoking and hypertension are important risk factors for CAD. Some previous studies showed a positive association between baldness and CAD when controlling for CAD risk factors [11-13]. However, some other studies showed no association, but these did not control for CAD risk factors [14-16]. The results of the two cohort studies [17,18] were inconclusive. In 2001, Rebora et al. [17] reviewed 24 articles in the literature from 1954 to 1999, as provided by MEDLINE. Baldness has no coincide with androgenetic alopecia in several of the articles examined, which makes it difficult to settle the issue. Subjects who develop baldness before their 30s may have a higher risk for CAD than other men, and there may be individuals with early-onset androgenetic alopecia who also present with unusually elevated dihydrotestosterone–testosterone ratios.

Interestingly, Mansouri et al. [18] found that an association between androgenetic alopecia and CAD in women. Their study was carried out in 106 women under age 55 years who underwent coronary arteriography to diagnose CAD. The correlation of androgenetic alopecia and CAD, androgenetic alopecia, a previous history of myocardial infarction, and greying of hair and CAD were statistically significant after adjustment of data for differences in age [18]. In our research, demonstrated that CAD might be increased in patients with baldness, but the severity of CAD does not increase in this patient. Although the exact pathophysiology of CAD in the bald patients remains unclear, some hypotheses are now suggested. First, the risk for CAD in bald individuals may reflect the interaction between balding and underlying major risk factors for CAD. In other words, the association between androgenic alopecia and insulin resistance, metabolic syndrome, and hypertension has been previously shown [19-21]. However, in our study, the impact of alopecia on CAD risk remains significant after adjusting such underlying risk factors. Second, it is now suggested that the association between androgenic alopecia and CAD may be mediated by flaring inflammatory processes and also increased peripheral sensitivity to androgens. It has been demonstrated that an inflammatory state can elevate the levels of inflammatory cytokines in the arterial walls and hair follicles [22,23]. High-sensitivity C-reactive protein is a marker of inflammation and also a good predictor of future cardiovascular disease so that chronic inflammation could be related to both CAD and baldness [24]. Male pattern baldness might be caused by increased peripheral sensitivity to androgens since bald men show an increase of androgen receptors in the scalp34 and have higher serum levels of both total and free testosterone [25]. Therefore, it seems that a combination of inflammatory processes, hypersensitivity to androgens along with triggering role of traditional CAD risk factors can affect susceptible individuals to CAD creation and progression.

## Conclusion

 Our study could successfully demonstrate an association between baldness and increased risk for CAD. Because we observed no significant association between baldness and CAD risk factors, this causality may be independent to other classic risk factors for CAD. Although we could not show the relation of CAD and its severity with balding and its grade, this lack of association may be caused by out potential limitations, such as considering only the number of involved coronary vessels as the indicator for CAD severity or used form grading system for baldness. In this regard, the critical role of genetic and racial background should not be ignored.

## Conflict of Interest

 The authors declare no potential conflicts of interest.

**Table 1:** Classification of Cardiovascular Risk Factors

**Classic cardiovascular risk factors**	**Number**	**Percent **
**Current or previous smoker**	42	40
**Opium user**	21	20
**Alcohol user**	10	6.7
**Diabetic**	25	24.
**Hypertensive**	41	39

**Table 2:** Coronary Angiography Reports

**Coronary angiography reports**	**Number**	**Percent **
**Normal coronary condition**	20	19
**Minimal CAD**	24	22.9
**Single-vessel disease**	15	15.2
**Two-vessel disease**	23	21
**Three-vessel disease**	18	17.1
**Left main lesion**	5	4.8

**Table 3:** Baldness Grading

**Grade of baldness**	**Number**	**Percent**
**Had no evidence**	51	48.6
**Class II**	16	15.2
**Class III**	8	7.6
**Class IV**	12	11.4
**class V**	7	6.7
**class VI**	7	6.7
**class VII**	4	3.8

**Table 4:** Comparing Baseline Parameters Between the Groups with and without Baldness

**Variables**	**With baldness** **(n=54)**	**Without baldness** **(n=51)**	**P-value**
**Age, year ( Mean±SD )**	62.24 ± 13.11	54.43 ± 12.76	0.003
**Body mass index, kg/m** ^2^	25.77 ± 2.92	26.69 ± 2.30	0.078
**Occupational status, n (%)**			
Army-related	35 (64.8)	27 (52.9)	0.357
Employed	13 (24.1)	15 (29.4)	
Self-employed	5 (9.3)	9 (17.6)	
Unemployed	1 (1.9)	0 (0.0)	
**Smoking, n (%)**	20 (37.0)	22 (43.1)	0.451
**Opium use, n (%)**	8 (14.8)	13 (25.5)	0.172
**Alcohol use, n (%)**	4 (7.4)	3 (5.9)	0.754
**Hypertension, n (%)**	23 (42.6)	18 (35.3)	0.434
**Diabetes mellitus, n (%)**	16 (29.6)	11 (21.6)	0.345
**History of baldness, n (%)**	26 (48.1)	4 (7.8)	<0.001

**Table 5:** Cardiovascular Status Among the Groups with and without Baldness

**Variables**	**With baldness** **(n=54)**	**Without baldness** **(n=51)**	**P-value**
**Presence of CAD**	48 (88.9)	37 (72.5)	0.033
**Severity of CAD, n( %)**			
None	6 (11.1)	14 (27.5)	0.291
Minimal CAD	13 (24.1)	11 (21.6)	
Single-vessel	8 (14.8)	8 (15.7)	
Two-vessel	12 (22.2)	10 (19.6)	
Three-vessel	11 (20.4)	7 (13.7)	
Left main lesion	4 (7.4)	1 (2.0)	
Left ventricular ejection fraction	42.30 ±11.16	46.24 ± 12.09	0.046

**Table 6:** Multivariable Logistic Regression Modeling to Assess the Value of Baldness for Predicting CAD

**Variables**	**B**	**SE**	**Wald**	**P-value**	**OR**
**Baldness **	1.007	0.726	1.924	0.046	3.037
**Age **	-0.043	0.029	2.183	0.140	0.958
**Body mass index**	-0.226	0.132	2.914	0.088	0.798
**Smoking **	0.599	0.666	0.808	0.369	1.820
**Opium use**	0.957	1.002	0.912	0.340	2.604
**Alcohol use**	0.691	1.325	0.272	0.602	1.996
**Diabetes mellitus**	1.869	0.901	4.299	0.038	6.480
**Hypertension **	1.542	0.691	4.977	0.026	4.676
**History of baldness**	0.591	0.993	0.354	0.552	1.806
**Constant**	-5.957	5.932	1.008	0.315	0.003
